# Identification of novel miRNAs potentially involved in the pathogenesis of adult T-cell leukemia/lymphoma using WGCNA followed by RT-qPCR test of hub genes

**DOI:** 10.1186/s13027-023-00492-0

**Published:** 2023-02-25

**Authors:** Ali Shayeghpour, Mohammad-Moien Forghani-Ramandi, Setayesh Solouki, Amin Hosseini, Parastoo Hosseini, Sara Khodayar, Mahsa Hasani, Sepehr Aghajanian, Zeinab Siami, Mohadeseh Zarei Ghobadi, Sayed-Hamidreza Mozhgani

**Affiliations:** 1 School of Medicine, Alborz University of Medical Sciences, Karaj, Iran; 2 Department of Computer, Faculty of Engineering, Raja University, Qazvin, Iran; 3 Department of Virology, School of Public Health, Tehran University of Medical Sciences, Tehran, Iran; 4 Research Center for Clinical Virology, Tehran University of Medical Sciences, Tehran, Iran; 5 Department of Microbiology, School of Medicine, Alborz University of Medical Sciences, Karaj, Iran; 6 Department of Infectious Diseases, School of Medicine, Alborz University of Medical Sciences, Karaj, Iran; 7 Independent Researcher, Tehran, Iran; 8 Non-Communicable Disease Research Center, Alborz University of Medical Sciences, Karaj, Iran

**Keywords:** Adult T-cell lymphoma/leukemia, ATLL, HTLV-1, WGCNA, Network analysis, Quantitative real-time PCR

## Abstract

**Background:**

Adult T-cell Lymphoma/Leukemia (ATLL) is characterized by the malignant proliferation of T-cells in Human T-Lymphotropic Virus Type 1 and a high mortality rate. Considering the emerging roles of microRNAs (miRNAs) in various malignancies, the analysis of high-throughput miRNA data employing computational algorithms helps to identify potential biomarkers.

**Methods:**

Weighted gene co-expression network analysis was utilized to analyze miRNA microarray data from ATLL and healthy uninfected samples. To identify miRNAs involved in the progression of ATLL, module preservation analysis was used. Subsequently, based on the target genes of the identified miRNAs, the STRING database was employed to construct protein–protein interaction networks (PPIN). Real-time quantitative PCR was also performed to validate the expression of identified hub genes in the PPIN network.

**Results:**

After constructing co-expression modules and then performing module preservation analysis, four out of 15 modules were determined as ATLL-specific modules. Next, the hub miRNA including hsa-miR-18a-3p, has-miR-187-5p, hsa-miR-196a-3p, and hsa-miR-346 were found as hub miRNAs. The protein–protein interaction networks were constructed for the target genes of each hub miRNA and hub genes were identified. Among them, UBB, RPS15A, and KMT2D were validated by Reverse-transcriptase PCR in ATLL patients.

**Conclusion:**

The results of the network analysis of miRNAs and their target genes revealed the major players in the pathogenesis of ATLL. Further studies are required to confirm the role of these molecular factors and to discover their potential benefits as treatment targets and diagnostic biomarkers.

**Supplementary Information:**

The online version contains supplementary material available at 10.1186/s13027-023-00492-0.

## Background

Adult T-cell lymphoma/leukemia (ATLL) affects the mature CD_4_ and CD_25_ lymphocyte lineage and has one of the poorest prognosis among hematologic malignancies [1]. The incidence of ATLL in the United States is about five individuals out of one million; however, it varies in diverse regions, with the incidence increasing up to about 27 in 100,000 in endemic zones [2]. According to the Shimoyama criteria, ATLL is divided into acute, chronic, lymphomatous, and smoldering subtypes [3]. The disease primarily occurs in the fifth and sixth decades of life [4] with poor response to conventional treatments [5]. Acute and lymphomatous types can shorten median survival to 6.2 and 10.2 months, respectively [4].

Human T-lymphotropic virus type 1 (HTLV-1) is a retrovirus and the causative agent of ATLL and a progressive chronic neurologic disorder, HTLV-1-associated myelopathy/tropical spastic paraparesis (HAM/TSP), which results in progressive weakness of the lower extremities and significant morbidity [6–8]. There is a 4–7% risk of developing ATLL in HTLV-1-infected individuals [9]. Its genome contains tax and Hbz, two regulatory genes [10, 11]. Hbz is encoded from the antisense strand of the genome [12]. Tax and Hbz play key roles in the formation of persistent infection and initiation of T cell oncogenesis. Tax interacts with several genes and signaling pathways of an infected host like nuclear factor-kB (NF-κB) pathway, Rat sarcoma virus (RAS) signaling pathway, and the mammalian target of rapamycin (mTOR) pathway [11]. Despite a large number of studies and promising discoveries on the topic, the exact molecular mechanisms responsible for HTLV-1-related tumorigenesis are not well known [13]. Yet, there are several studies searching for anomalies in the area of epigenetics [14–16]. MicroRNAs (miRNAs) belong to a wide group of non-coding RNAs that mainly govern gene expression [17]. These small single-stranded RNAs also regulate a broad spectrum of biological processes through mRNA silencing and degradation [18]. The upregulation of miRNAs in cancer cells may lead to carcinogenesis by interdicting tumor suppressor genes. These miRNAs are known as oncogenic miRNAs (oncomiRs) [19]. On the other hand, there are miRNAs with tumor suppressor function that can result in the prevention of cancer progression by interfering with the expression of proto-oncogenes [20]. These miRNAs are identified as tumor suppressor miRNAs [21]. So far, miRNAs have been mentioned to be dysregulated in HTLV-1 infected cells, most likely influencing important signaling pathways such as pathways in cancer, WNT signaling pathway, MAPK signaling pathway, and other important pathways involved in carcinogenesis [10].

Owing to the innovations in methods of extracting genome-wide assays for biological samples in recent years, it is now possible to investigate cellular physiology and the pathophysiology of diseases in a systematic manner. One strategy in biological system analysis is to organize and study genes based on their interactions [22]. In this regard, several approaches may be utilized [23–27]. This study used weighted gene co-expression network analysis (WGCNA) for the purpose of classifying miRNAs into modules based on their correlations and then surveying the co-expressed miRNAs in each module. It helps to find major miRNAs with high connectivity with others that probably have critical functions in the progression of the disease.

Considering ATLL’s rapid progression and the fact that it is refractory to conventional chemotherapy [28], there is a great need to have a better understanding of the pathogenesis by finding factors that have key roles in the development of the disease. The present study investigates the co-expressed dysregulated microRNAs (miRNAs) involved in the ATLL pathogenesis using weighted gene co-expression analysis. A number of target genes of the affected miRNAs are also studied to delineate and confirm the signaling pathways that may be implicated in the development and progression of ATLL.

## Methods

### Microarray dataset and data preprocessing

**Fig. 1:**
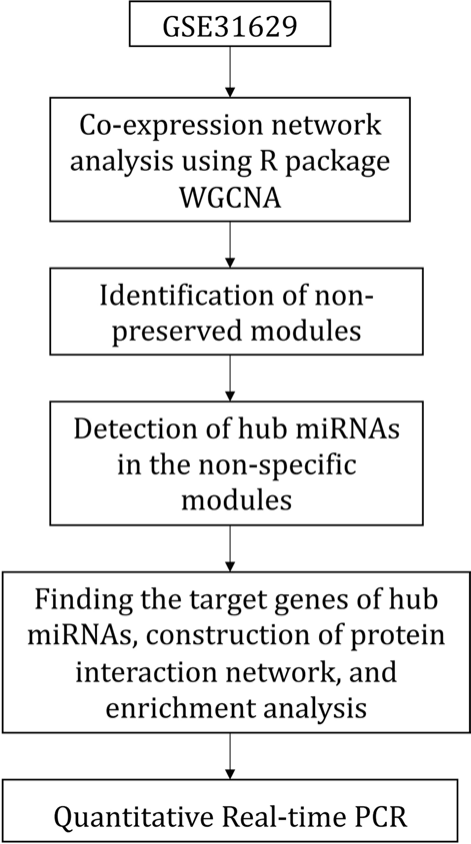
The flowchart of the methodology

Under the accession number GSE31629 [29], microRNA expression dataset of 62 samples including 40 ATLL patients and 20 healthy individuals was retrieved from the gene expression omnibus (GEO) database [30]. The data derived from peripheral blood mononuclear cells (PBMCs) and CD4+ T cells were normalized with quantile normalization. Missing values were handled using the "goodSamplesGenes" function from the WGCNA package, version 1.71 [31, 32]. Subsequently, sample clustering was used to identify outliers. The summary of the methodology is presented in Fig. 1.

### Construction of co-expression network and module detection

The weighted co-expression network was constructed using the WGCNA package” version 1.71 in the R environment [33]. To do so, an adjacency matrix was built using the following formula:

$${\text{a}}_{{{\text{ij}}}} = \left| {0.5 + 0.5*{\text{cor}}\left( {{\text{X}}_{{\text{i}}} ,{\text{X}}_{{\text{j}}} } \right)} \right|^{\beta }$$

where β is the exponent and X_i_ and X_j_ are the expression values of miRNAi and miRNAj, respectively. The soft-thresholding approach was applied to obtain a scale-free topology. Using the “picksoftthreshold” function of the WGCNA package, β = 4 was determined to be the optimal exponent value.

In the next step, the topological overlap measure (TOM) was determined as the measure of miRNAs connectivity in the network. Eventually, the co-expressed gene groups (modules) were detected by hierarchical clustering with parameters of *minModuleSize* = 15, and a threshold of 0.25 was chosen to merge the close modules.

### Identification of non-preserved modules

The non-preserved modules of ATLL in the healthy samples were determined through module preservation analysis. For this purpose, the “modulePreservation” function [34] and permutation-based statistics were applied to measure medianRank and Z_summary_ scores [35, 36]. Z_summary_ is the average of Z-scores calculated for connectivity and density measures [37]. Herein, a module with Z_summary_ < 2 and medianRank > 12 was considered a non-preserved module.

### Finding hub miRNAs and their target genes

To find the hub miRNA in each non-specific module, the function of “chooseTopHubInEachModule” in the WGCNA package was utilized. In order to find the experimentally validated genes for the mentioned hub miRNAs, the miRTarBase database was explored. miRTarBase is a collection of nearly 20 million experimentally validated miRNA–target interactions (MTIs) among 4630 miRNAs and 27,172 mRNAs [38].

### Protein–protein interactions (PPIs) and enrichment analysis

The association between proteins was determined using the STRING database [39]. Due to the large size of PPINs, degree analysis was implemented to select genes with more connectivity which are also known as hub genes. The degree of a protein is its connection number to other proteins in the network. To survey the biological process of the identified target genes, the Enrichr webtool was utilized [40].

### Sample preparation

This study was conducted on 10 ATLL samples from patients who had been admitted to the Shariati Hospital, Tehran, Iran, and 10 normal samples from blood donors who were referred to the Blood Transfusion Organization of Alborz. Peripheral blood mononucleolar cells (PBMCs) were isolated from EDTA-treated blood samples using a Ficoll-Paque density gradient (Cederline corporation, Canada). Total RNA was extracted from PBMCs with RNA Extraction RNJia Kit-ROJE Technology and the cDNA was synthesized using the RT-ROSET kit (ROJE, Iran). This study was approved by the Ethical Committee of Biomedical Research at Alborz University of Medical Sciences (IR.ABZUMS.REC.1399.342).

### Quantitative real-time PCR

To confirm the HTLV-1 infection in the ATLL patients, the LTR and HBZ genes’ expressions were evaluated by PCR and subsequent agarose gel electrophoresis [41, 42]. Quantitative Real-Time PCR was performed on cDNAs utilizing specific primers and SYBR Green-based RT-qPCR (TaKaRa, Otsu, Japan). To measure the expression rate of UBB, RPS15A, and KMT2D genes in ATLL and normal groups, the PCR was done on a Rotor-Gene Q-6000 machine (Qiagen, Germany) following the manufacturer’s instructions. According to five 5-point standard curves, that had been already prepared for target and reference genes, the gene expression of UBB, RPS15A, KMT2D, and RPLP0 (reference gene) were analyzed. The normalized gene expression was calculated as follows: Normalized index = copy number of the gene of interest/copy number of the reference gene.

### Statistical analysis

The Mann–Whitney U Test was used to compare gene expression levels between the normal and ATLL groups using GraphPad Prism software v8 (GraphPad Software, Inc., San Diego, CA, USA). The data is displayed as mean ± standard error of the mean (SEM). The results were considered statistically significant if the *P*-value < 0.05.

## Results

### Construction of weighted gene co-expression network

**Fig. 2:**
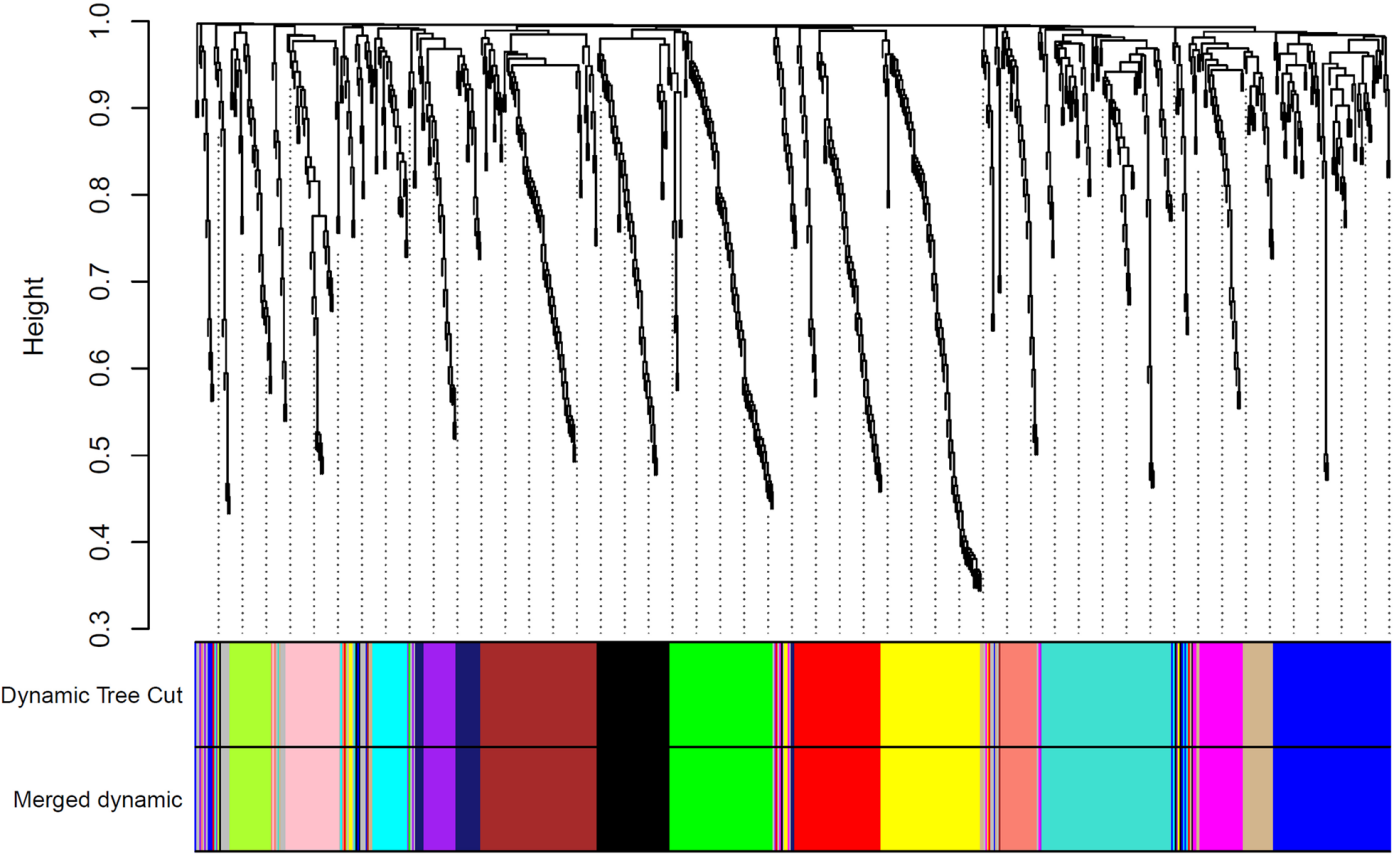
Dendrogram of clustered genes based on (1-TOM). The colors reveal the cluster (module) membership acquired before and after merging the modules. A total of 14 modules were identified after merging

To identify the co-expressed miRNAs, the calculation of adjacency and TOM matrices, construction of hierarchical clustering, and finally merging of close clusters were performed. As a result, 15 modules were identified. Figure 2 displays the cluster dendrogram and modules before and after merging the clusters. Each unique color specifies an inimitable module. A list of co-expressed miRNAs in each module and their connectivity (degree) scores are mentioned in Additional file 1.

### Determination of specific modules for the ATLL samples

**Fig. 3:**
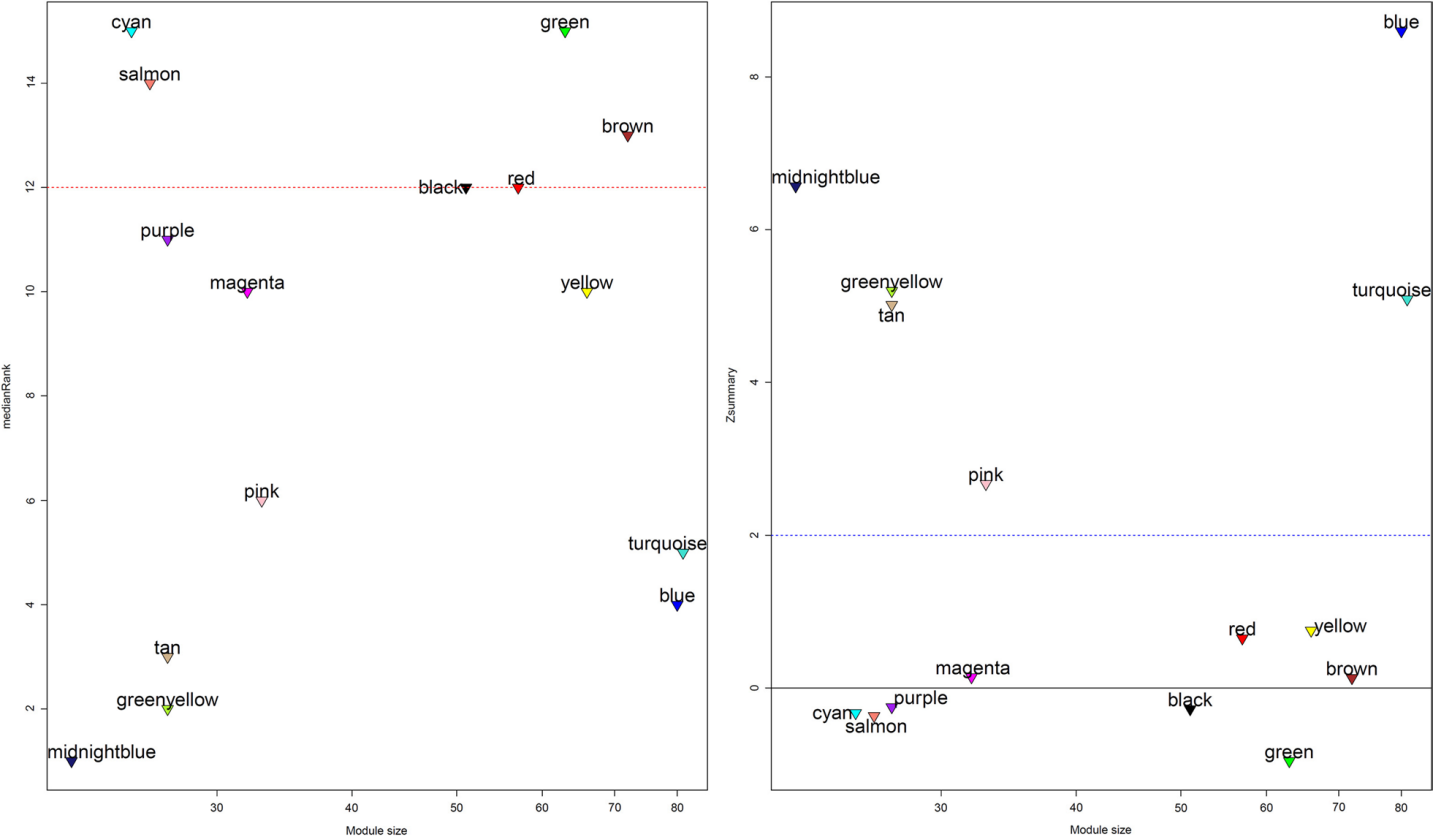
The medianRank (plot on the left) and Z_summary_ (plot on the right) against module size specifies the non-preserved modules (Z_summary_ < 2 and medianRank > 12)

To detect the specific non-preserved modules of ATLL samples, the medianRank and Z_summary_ scores were calculated for each module. The Z_summary_ < 2 and medianRank > 12 were considered as criteria to find the non-preserved modules. Among 15 identified modules, four modules including green, salmon, cyan, and brown modules were identified as specific modules for ATLL (Fig. 3).

### Finding hub miRNA and their target genes

As the result, “chooseTopHubInEachModule” function identified hsa-miR-346, hsa-miR-187-5p, hsa-miR-196a-3p, and hsa-miR-18a-3p as hubs in the green, salmon, cyan, and brown modules, respectively. Target genes of each miRNA were obtained from miRTarBase for further analysis. The target genes are mentioned in Additional file 2.

### PPINs and enrichment analysis

**Fig. 4:**
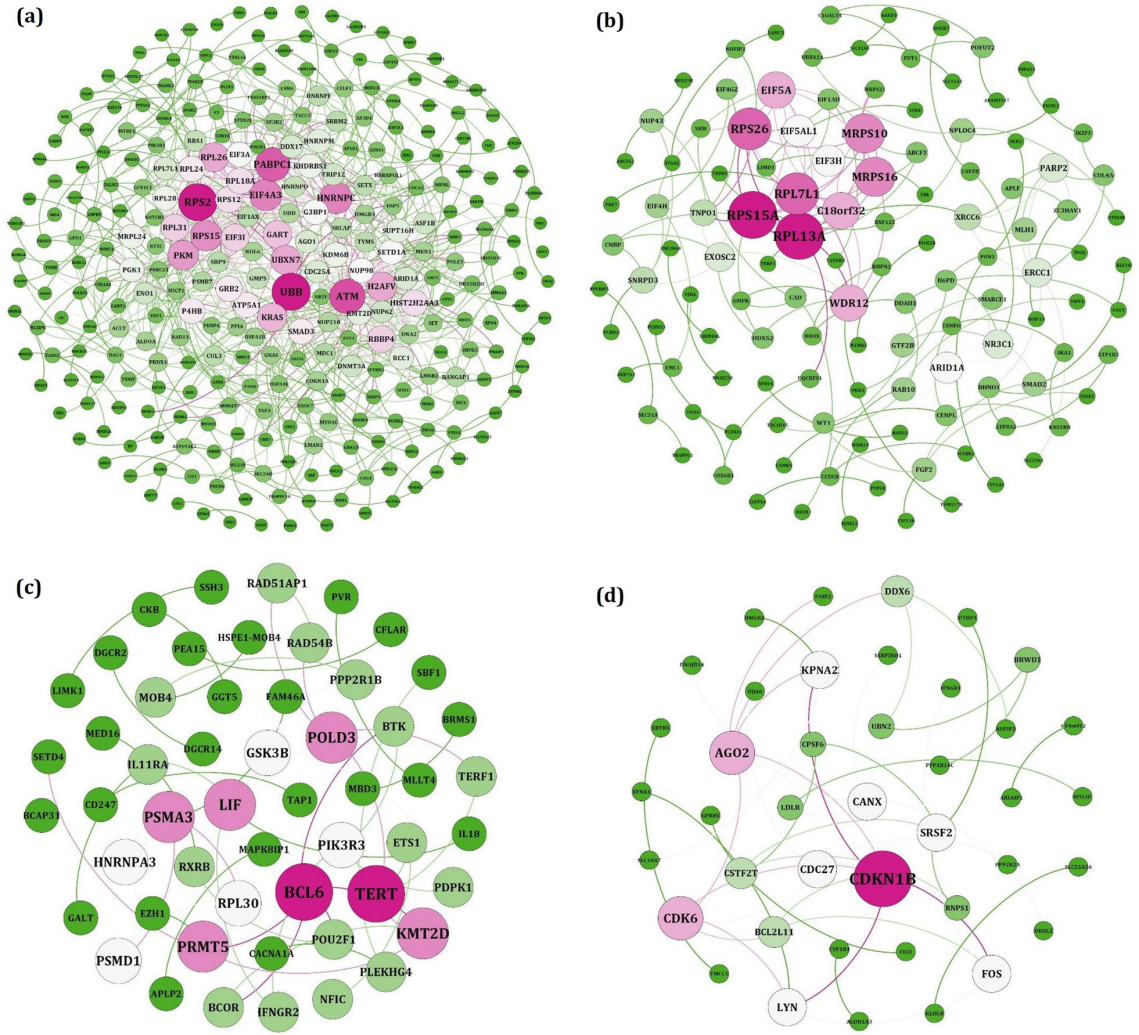
The protein–protein networks among the target genes of **a** hsa-miR-18a-3p, **b** hsa-miR-196a-3p, **c** hsa-miR-346 and **d** hsa-miR-187-5p. The color and the size of nodes is related to their degree (red color and bigger size indicate higher values of degree)

**Fig. 5:**
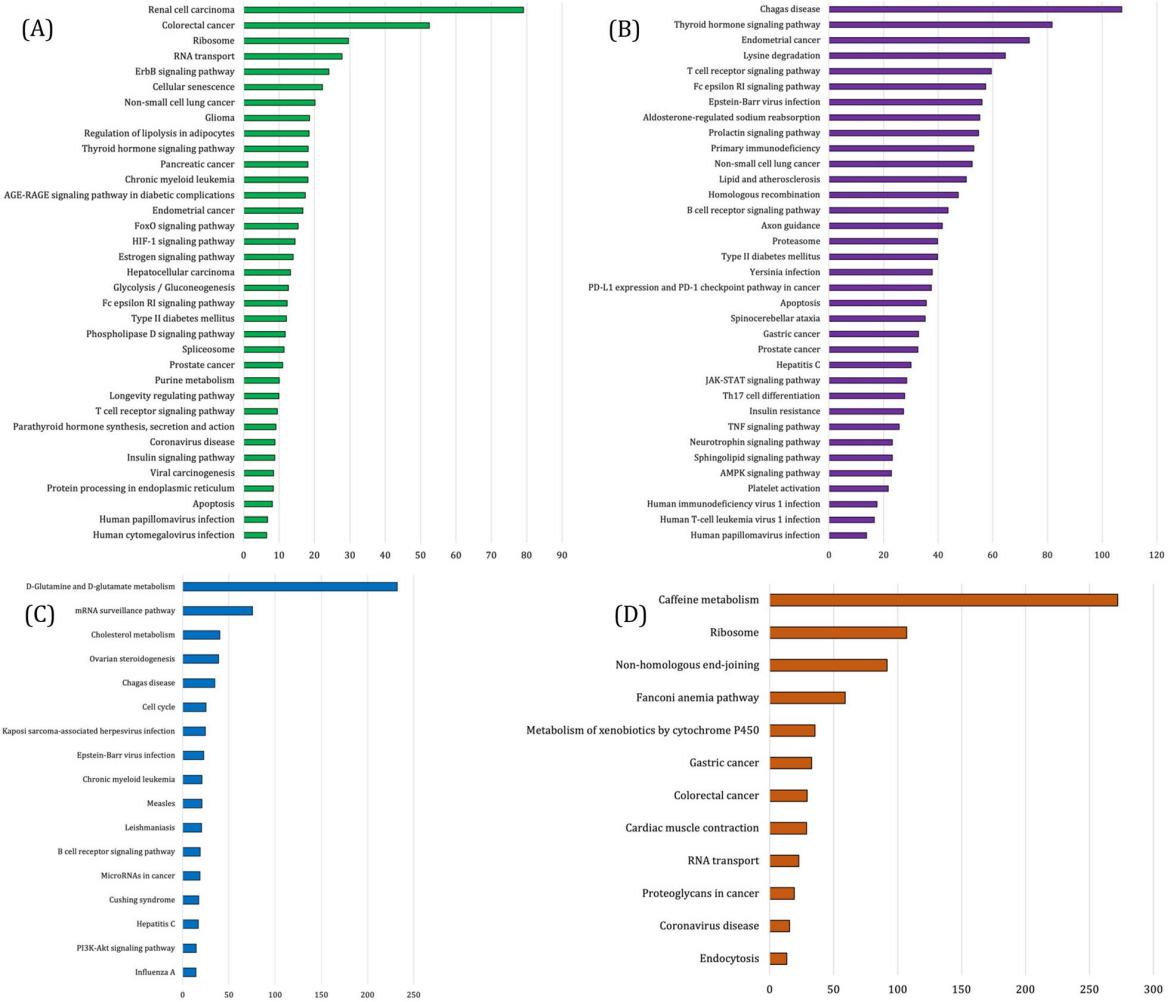
Significant KEGG pathways enrichment results of **A** hsa-miR-18a-3p, **B** hsa-miR-346, **C** hsa-miR-187-5p and **D** hsa-miR-196-3p target genes. All of the pathways are statically significant (*P* value < 0.05) and are sorted based on the combined scores provided by Enrichr

The PPINs between the proteins were constructed by submitting the target genes of hub miRNAs belonging to the specific modules in the STRING database (Fig. 4). Further network analysis revealed the degree of each protein. The protein with a higher degree is demonstrated as red. Based on this analysis, UBB and RPS2 (hsa-miR-18a-3p); RPL13A and RPS15A (hsa-miR-196a-3p); BCL6, TERT, KMT2D (hsa-miR-346); CDKN1B and AGO2 (hsa-miR-187-5p) were identified as hub proteins (Table 1). Subsequently, biological process enrichment was performed to find the function of each hub genes. The enrichment results are presented in Fig. 5.

**Table 1:** List of hub miRNAs and target genes involved in ATLL progression

Hub miRNAs	Hub target genes
hsa-miR-18a-3p	UBB and RPS2
hsa-miR-196a-3p	RPL13A and RPS15A
hsa-miR-346	BCL6, TERT, KMT2D
hsa-miR-187-5p	CDKN1B and AGO2

### Lower expression of UBB, RPS14A and KMT2D in ATLL patients compared to the normal control

**Fig. 6:**
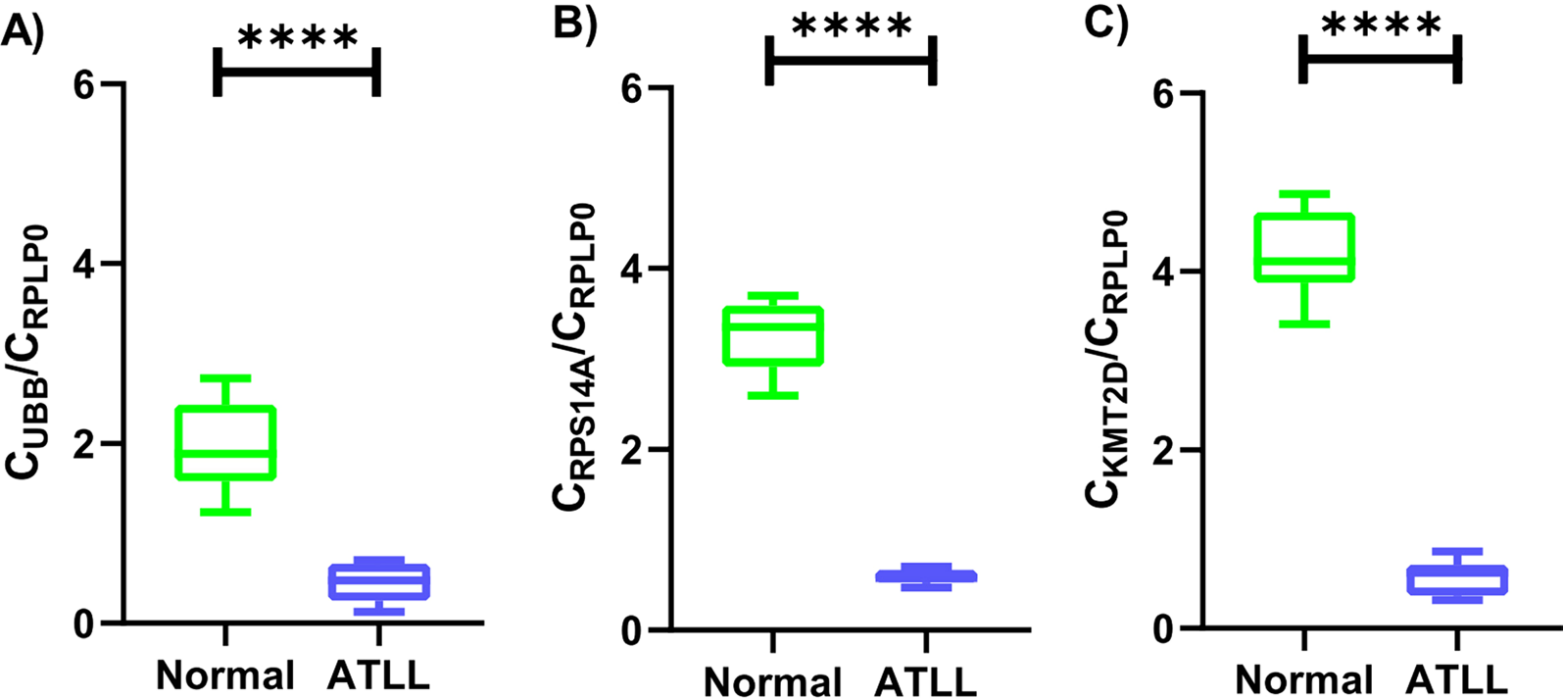
Quantitative results of RT-PCR show significant downregulation (*P*-value < 0.0001) of **A** UBB, **B** RPS15A and **C** KMT2D

The remarkable association between the highlighted miRNAs and pathways related to malignancies, immunity, and viral infections led us to examine the gene expression of some of the hub genes that their dysregulation in ATLL patients was never reported before. The results demonstrated significant downregulation of all three genes (*P*-value < 0.0001) in ATLL patients versus healthy controls (Fig. 6).

UBB’s expression rate in the ATLL group (0.4484 ± 0.06539) was lower than in the normal group (1.952 ± 0.1515) and the difference was statistically significant with *P*-value < 0.0001. A significantly lower expression of RPS14A in the ATLL patients (0.5884 ± 0.02465) compared to the normal group (3.260 ± 0.1204) was also observed (*P*-value < 0.0001). The expression of KMT2D in the ATLL cases was 0.5740 ± 0.05896 and in the normal control was 4.204 ± 0.1548, which shows a statistically significant lower expression in the ATLL patients than in the normal cases (*P*-value < 0.0001).

## Discussion

Weighted gene co-expression analysis is a robust method for identifying expression patterns of diseases in a variety of tissues across different pathological states. It is reasonable to expect that the specific co-expression patterns (or modules) for patient samples are likely associated with the underlying pathophysiological pathways and processes. As a corollary, module preservation analysis can be utilized to identify condition-specific or non-preserved modules.

In the present study, a number of hitherto unexplored miRNAs and genes were identified as possibly implicated in ATLL pathogenesis. All genes are miRNAs driver genes that have been found through exploring miRTarBase database. Module preservation analysis disclosed 4 specific modules for ATLL. The hub miRNAs in these modules have a substantial correlation with numerous cancer pathways in an enrichment assessment of their target genes. Subsequent in vitro analysis of the identified miRNAs' target genes demonstrated decreased expression of KMT2D and its potentially important role in ATLL pathogenesis.

It is believed that miR-346 may be involved in both inflammatory and metabolic processes [43]; however, additional research has shown that miR-346 may also have a role in the development of malignancies of the breast, cervix, and lung, as well as follicular thyroid, cutaneous squamous cell, and nasopharyngeal carcinomas [43–49]. The enrichment analysis of its target genes also indicated that miR-346 is deeply associated with pathways related to viral infections and malignancies; therefore, we further studied its target hub gene, KMT2D. Lysine Methyltransferase 2D, or KMT2D, is a member of a complex of proteins associated with Set1 (COMPASS), which enables gene transcription through H3K4 methylation [50]. KMT2D plays contradictory roles in different types of malignancies, some studies stated it has pro-tumorigenic functions [51], whereas others reported tumor-suppressive characteristics [50–53]. As a tumor suppressor gene, KMT2D is known to activate Bcl6 and Sirt1 in addition to Per2, resulting in inhibitory effects on Notch and K-Ras pathways [51]. It is good to notice that Bcl6 is also a miR-346 target hub gene. Loss of KMT2D was shown to assist cell growth related to EZH1/2 in pre-tumorigenic EBV + B cell lymphoblastoid cells [54]. Our results show significant downregulation of KMT2D in ATLL patients versus healthy controls. Put it all together, these shreds of evidence are highly suggestive of its involvement in the pathogenesis of the disease, but considering its antithetical effects on cancers, more studies are needed to clarify the exact influence of KMT2D on ATLL.

In recent years miR-187 has attracted the attention of more researchers due to discoveries about its roles in various cancer types. miR-187 is likely to be involved in many cancer-related processes and characteristics such as sensitivity to drugs, proliferation, apoptosis, invasion, and migration through its regulatory effects on its targets, namely FOXA2, CRMP1, MAD2L2, STOML2, BCL6, PTRF, CYP1B1, FGF9, MAPK12, MAPK7, Bcl-2, IGF-1R [55]. Its dysregulation has been observed in many malignancies like osteosarcoma, male genitourinary tumors, prostate cancer, bladder cancer, clear cell renal cell carcinoma, colorectal cancer, hepatocellular carcinoma, oral squamous cell carcinoma, cervical cancer, ovarian cancer, breast cancer, lymphoblastic leukemia, and diffuse large b-cell lymphoma [55–73]. To our knowledge, the relationship between Adult T-Cell Leukemia/Lymphoma and miR-187 was never reported before. Our high-throughput analysis of miRNAs proposes the involvement of miR-187 in the pathogenesis of ATLL. Considering the notable effects of miR-187 on a high variety of malignancies, it seems necessary to investigate the miR-187 actions in ATLL patients.miR-18a-3p was highlighted as a tumor-suppressive agent through its inhibitory effect on K-Ras [74]. It was also reported that miR-18a-3p has a regulatory effect on glycolysis/gluconeogenesis and focal adhesion in the uterus, lung, liver, and kidney malignancies [75]. There are studies presenting pieces of evidence that show miR-18a-3p is related to nasopharyngeal and hepatocellular carcinoma, breast cancer, and glioma [76–79]. Accordingly, to do an additional survey, Ubiquitin B (UBB) was selected as it was one of the hub genes in PPIN which is also known to play key roles in basic cellular functions. UBB is one of four Ubiquitin encoding genes that is involved in the dysregulation of fundamental processes like cellular proliferation, apoptosis, and responses to DNA damage [80]. Several relationships between Ubiquitin and various types of cancers have been reported such as gynecological cancer, hepatocellular carcinoma, colorectal cancer, neuroblastoma, Esophageal Squamous Cell Carcinoma, Pediatric Medulloblastoma, seminoma, and lung cancer [80–82]. According to the downregulation of UBB in the RT-PCR study, the possibility of its pathogenic actions in ATLL can be ruled out.miR-196a was shown to play roles in inflammation, embryonic development, and various cancers such as pancreatic adenocarcinoma, breast cancer, leukemia, esophageal adenocarcinoma, and colorectal cancer [83]. Ribosomal protein S15A (RPS15A) has been highlighted as a candidate participating in ATLL tumorigenesis. It belongs to the 40S subunit of ribosome [84]. RPS15A was reported to be potentially involved in hepatocellular carcinoma, progression of breast cancer, lung adenocarcinoma, prostate cancer, glioblastoma, colorectal cancer, and acute myeloid leukemia as an oncogene [84–87]. RPS15A was also shown to be downregulated in ATLL patients in the PCR study, which rejects our hypothesis.

Our study have some limitations. We did not find a large miRNA dataset in databases. However, it is better that the mentioned analysis perform in a larger dataset if in it provides in future. The genes and also miRNAs should be validated in a large cohort.

## Conclusion

The weighted miRNA-co-expression analysis, network analysis of the target genes, enrichment analysis, and comprehensive literature review highlighted hsa-miR-18a, has-miR-187, hsa-miR-196a-3p, and hsa-miR-346 as four new miRNAs that are strongly suspected to be involved in the pathogenesis of ATLL. Further investigation is needed to assess the utility of these findings and their values as reliable biomarkers and/or therapeutic targets. The expression levels of the three hub genes of the identified miRNAs target genes, namely, UBB, RPS15A, and KMT2D also imply the significant role of the miRNAs in maintaining the dysregulated pattern of gene expression in ATLL. Likely, the downregulation of KMT2D plays an important role in ATLL carcinogenesis, but its controversial effects on different cancer types preclude a definite conclusion regarding its role.

## Supplementary Information

**Additional file 1.** List of co-expressed miRNAs and their connectivity scores in each module.

**Additional file 2.** The target genes of non-preserved modules.

## Data Availability

The datasets used and/or analyzed during the current study are available from the corresponding author on reasonable request.
